# Formulation and Therapeutic Evaluation of Isoxsuprine-Loaded Nanoparticles against Diabetes-Associated Stroke

**DOI:** 10.3390/pharmaceutics15092242

**Published:** 2023-08-30

**Authors:** Heba A. Abou-Taleb, Basmah Nasser Aldosari, Randa Mohammed Zaki, Obaid Afzal, Alaa S. Tulbah, Mary Girgis Shahataa, Fatma I. Abo El-Ela, Heba F. Salem, Amr Gamal Fouad

**Affiliations:** 1 Department of Pharmaceutics and Industrial Pharmacy, Faculty of Pharmacy, Merit University (MUE), Sohag 1646080, Egypt; heba.ahmed@merit.edu.eg; 2 Department of Pharmaceutics, College of Pharmacy, King Saud University, P.O. Box 2457, Riyadh 11451, Saudi Arabia; baldosari@ksu.edu.sa; 3 Department of Pharmaceutics, College of Pharmacy, Prince Sattam Bin Abdulaziz University, P.O. Box 173, Al-Kharj 11942, Saudi Arabia; randazaki439@yahoo.com; 4 Department of Pharmaceutics and Industrial Pharmacy, College of Pharmacy, Beni-Suef University, Beni-Suef 62514, Egypt; 5 Department of Pharmaceutical Chemistry, College of Pharmacy, Prince Sattam Bin Abdulaziz University, P.O. Box 173, Al-Kharj 11942, Saudi Arabia; o.akram@psau.edu.sa; 6 Department of Pharmaceutics, College of Pharmacy, Umm Al Qura University, Makkah 21421, Saudi Arabia; astulbah@uqu.edu.sa; 7 Department of Pharmacology, Faculty of Medicine, Beni-Suef University, Beni-Suef 62511, Egypt; mary.samaan@med.bsu.edu.eg; 8 Department of Pharmacology, Faculty of Veterinary Medicine, Beni-Suef University, Beni-Suef 62511, Egypt; fatma.aboel3la@vet.bsu.edu.eg; 9 Department of Pharmaceutics and Industrial Pharmacy, Faculty of Pharmacy, Beni-Suef University, Beni-Suef 62514, Egypt; amr_g@pharm.bsu.edu.eg

**Keywords:** stroke, diabetes mellitus, isoxsuprine, liposomes, propylene glycol

## Abstract

Ischemic stroke is the second-leading cause of death. Hyperglycemia, which is characteristic of diabetes mellitus, contributes to the development of endothelial dysfunction and increases the risk of stroke. Isoxsuprine is an efficient beta-adrenergic agonist that improves blood flow to the ischemic aria and stops the infarct core from growing. However, low bioavailability, a short biological half-life, and first-pass hepatic metabolism reduce the therapeutic efficacy of oral isoxsuprine. Therefore, the authors focused on developing isoxsuprine-loaded liposomes containing ethanol and propylene glycol (ILEP) formulation as nasal drops for the treatment of ischemic stroke in diabetic patients. Different ILEP formulations were optimized using Design Expert software, and the selected formulation was examined in vivo for its anti-stroke effect using a rat model of diabetes and stroke. The optimized ILEP, composed of 15% propylene glycol, 0.16% cholesterol, 10% ethanol, and 3.29% phospholipid, improved the sustainability, permeation, and targeting of isoxsuprine. Furthermore, the in vivo studies verified the improved neurological behavior and decreased dead shrunken neurons and vascular congestion of the rats treated with the optimized ILEP formulation, demonstrating its anti-stroke activity. In conclusion, our study found that treatment with an optimized ILEP formulation prevented the initiation and severity of stroke, especially in diabetic patients.

## 1. Introduction

Ischemic stroke occurs when oxygen and nutrients are denied to brain tissue because of a lack of blood flow to that area of the brain [1,2]. Among developed-world populations, it ranks second in terms of mortality and third in terms of disability [1,2,3]. Hyperglycemia is a hallmark of diabetes mellitus, and it is linked to complications such as ischemic stroke [1,2,3]. Patients with diabetes have a much-increased chance of suffering an ischemic stroke [1,2,3]. Therefore, diabetic patients should be prescribed anti-stroke medicine for prevention to reduce their increased mortality risks. Multiple studies linked beta-adrenergic agonist therapy to improved outcomes for patients with cerebrovascular insufficiency and peripheral vascular disease [4,5,6]. Isoxsuprine (ISP) is an efficient beta-adrenergic agonist that improves blood flow to the vulnerable penumbral region during ischemia and stops the infarct core from growing [7,8,9]. Because of its ability to selectively inhibit the NMDA receptor in the NR2B subtype, it has been approved by the FDA as a promising neuroprotective agent [5,6]. ISP blocks α1-adrenoreceptor and so increases β2-agonism-mediated cerebral vasodilation and decreases vasoconstriction in ischemic injury [5,6]. ISP also improves glucose uptake, insulin secretion, and fat breakdown [10,11,12]. However, low bioavailability and first-pass hepatic metabolism reduce the therapeutic efficacy of oral ISP [13,14]. Despite its convenience, oral ISP is not well tolerated by patients because of its high dose frequency, short biological half-life, and rapid elimination [14,15].

The nasal route holds promise for the direct delivery of drugs to the brain and is an appealing alternative to other administration routes [16,17]. The high vasculature, lack of enzymes in the nasal cavity, and bypass of the liver’s first-pass metabolism make the nasal route an excellent choice for systemic drug delivery [16,17,18]. Nasal administration also appears to be an effective strategy for avoiding the blood–brain barrier and achieving cerebral delivery of drugs [17]. The rapid mucociliary clearance and the requirement for drug molecules to pass the nasal epithelium are two limitations of nasal drug absorption [16,17,18]. Lipid-based nanoparticles are seen as a promising option for addressing these limitations and improving the efficacy of nasal medication delivery. Liposomes, which are made up of cholesterol and phospholipids, are the most common type of nanoparticle because of their ability to distribute drugs in a targeted and controlled manner, thereby increasing the drug’s half-life and decreasing the dose frequency of the drug and improving patient compliance [19,20]. Phospholipids and cholesterol increase the viscosity of the formulations, which in turn reduces the mucociliary clearance [19,20]. However, liposomes have poor drug encapsulation efficiency, poor stability, and poor penetration [19,20,21,22]. Ethanol and propylene glycol have been introduced as penetration enhancers to improve the vesicular characteristics and permeability of liposomes [19,20,21,22]. The lipid bilayer of the nasal barrier is made less stiff by propylene glycol and ethanol, which also increases the barrier’s pliability, diffusion, and fluidity. The addition of propylene glycol and ethanol increases the liposomes’ negative charge, viscosity, and anti-hydrolysis characteristic, improving their penetration and stability [21,22]. With this study, the authors focused on developing ISP-loaded liposomes containing ethanol and propylene glycol (ILEP) formulation as nasal drops for the treatment of ischemic stroke in diabetic patients. The design of the ILEP formulation was obtained to enhance nasal penetration of ISP and maintain the ISP’s release to lengthen its biological half-life and reduce its elimination and dose frequency, thereby increasing bioavailability, efficacy, and patient compliance with ISP. Different ILEP formulations were optimized using Design Expert software, and the selected optimized formulation was examined in vivo for its anti-stroke effect using a rat model of diabetes and stroke.

## 2. Materials and Methods

### 2.1. Materials

Isoxsuprine was kindly donated by the Egyptian branch of Abbott Pharmaceuticals (Cairo, Egypt). Cholesterol and phospholipids were purchased from the Egyptian branch of Sigma-Aldrich (Agitech, Cairo, Egypt). Methanol, ethanol, propylene glycol, and chloroform were purchased from Cornell Lab (Cairo, Egypt).

### 2.2. Experimental Design

Design Expert software^®^ (version 12, StatEase Inc., Minneapolis, MN, USA) was used to generate a 3^3^ Box–Behnken design for the preparation of 15 formulations of ISP-loaded liposomes containing ethanol and propylene glycol (ILEP), with X_1_ representing the concentration of propylene glycol, X_2_ representing the concentration of phospholipid, and X_3_ representing the concentration of ethanol (Table 1 and Table 2). Entrapment efficiency (EE%) and vesicle size were used as dependent variables.

### 2.3. Preparation of CLEP Formulations

Preliminary experiments were conducted to assess if the presence of phospholipid and cholesterol had any impact on the absorption outcomes of ISP. This was achieved by analyzing the absorption spectra of plain liposomes containing ethanol and propylene glycol (PLEP) formulations as a blank formulation at a wavelength of 274 nm. Different ILEP and PLEP formulations were developed using the thin-film hydration technique [23]. A solution of phospholipid, propylene glycol, and cholesterol (0.16%) in chloroform/methanol was evaporated at 40 °C and 100 rpm under vacuum in a rotary evaporator (VV 2000, Burladingen, Germany) to produce a thin film of ILEP. The ILEP film was rehydrated in phosphate buffer (pH 6.8) containing ISP (10 mg) and ethanol at 60 rpm, and the resultant suspension was stored at 4 °C.

### 2.4. Characterization of ILEP Formulations

#### 2.4.1. Entrapment Efficiency Measurement

Unentrapped ISP was separated by spinning the ILEP formulation at 15,000 rpm for 1 h in a cooling centrifuge (SIGMA 3-30 K, Sigma, Steinheim, Germany). After collecting the ILEP pellets, they were mixed with phosphate buffer (pH 6.8) and placed in the refrigerator until analysis. We ensured that all of the ILEP pellets were successfully separated by recentrifuging the unentrapped ISP solution at 15,000 rpm for 2 h at 4 °C till a clear solution resulted [24,25,26]. Using a standard calibration curve and a UV–visible spectrophotometer set to a wavelength of 274 nm, we were able to calculate the concentration of unentrapped ISP using an indirect method [27,28]. After taking measurements in triplicate, we used the following formula to calculate the EE%:

(1) $$\mathrm{Entrapment}\;\mathrm{efficiency}\;\left(\%\right)=\left(\left(Ct\;-\;Cf\right)/Ct\right)\times 100$$

where *Ct* is the total amount of ISP, and *Cf* is the amount of free ISP.

#### 2.4.2. Vesicle Size Measurement

To determine the vesicle size of different ILEP formulations, a sample was diluted and investigated using dynamic light scattering on a piece of zeta sizer equipment (Malvern, Worcestershire, UK) [29].

### 2.5. Optimization of ILEP Formulations

Analysis of variance (ANOVA) in Design Expert software was used to analyze the results for each dependent variable, with criteria including lack of fit, F-value, and *p*-value utilized to establish the optimal model [30]. Applying criteria of maximum entrapment efficiency and minimum vesicle size allowed for optimization in accordance with the desirability index. The optimized formulation was prepared and tested in triplicate to verify the software’s calculated factors and predicted responses.

### 2.6. Characterization of the Optimized ILEP Formulation

#### 2.6.1. Differential Scanning Calorimetry (DSC)

The interaction between ISP and the ILEP-producing components was evaluated by analyzing the thermograms of ISP, cholesterol, phospholipid, and optimized ILEP with a differential scanning calorimeter (Shimadzu DSC-50, Kyoto, Japan) [20]. The DSC scan was performed with a nitrogen flow rate of 100 mL/min and temperatures ranging from 25 to 250 °C, at a rate of 5 °C/min.

#### 2.6.2. Transmission Electron Microscopy (TEM)

To examine the morphological characteristics of the optimized ILEP formulation, a transmission electron microscope (Tokyo, Japan) was used [31]. On a carbon-coated grid, we diluted some ILEP formulation and stained it with 2% phosphotungstic acid.

#### 2.6.3. Zeta Potential and Size Distribution

The stability of the ILEP can be determined by measuring the zeta potential, which is a quantitative evaluation of the strength of repulsive forces between vesicles [29]. The polydispersity index (PDI) was used to determine how evenly distributed and of a consistent vesicle size a given sample was [29]. Dynamic light scattering (Malvern, Worcestershire, UK) was used to evaluate the optimized ILEP formulation for zeta potential and PDI.

#### 2.6.4. In Vitro Release Study

Utilizing a diffusion method with dialysis bags (Mwt cut-off 12,000; Sigma-Aldrich, Cairo, Egypt), we compared the drug release from the optimized ILEP to that from free ISP [30]. To achieve the sink condition, the dialysis bags were filled with free ISP and optimized ILEP formulation (equivalent to 3 mg ISP), and phosphate buffer (20 mL, pH 6.8) was used as the medium of release inside a Hanson dissolution apparatus (Düsseldorf, Germany) at 37 °C and 100 rpm. Over a period of 24 h, three-milliliter samples were taken and replaced with fresh release medium to keep the sink condition constant at all times. At a wavelength of 274 nm, the ISP concentration in each sample was calculated using a UV–visible spectrophotometer. The cumulative percentage of ISP releases was calculated three times and presented as mean ± SD. 

#### 2.6.5. Ex Vivo Permeation Study

Using a diffusion membrane made from an epidermal layer of abdominal rat skin, we investigated the ex vivo permeation of free ISP and the optimized ILEP formulation. The experiment was approved by the ethics committee at Beni-Suef University in Egypt. Here, phosphate buffer (20 mL, pH 6.8) was used as the receptor medium inside a Hanson dissolution apparatus at 37 °C and 100 rpm [23]. Over a period of 24 h, three-milliliter samples were taken and replaced with fresh release medium to keep the sink condition constant at all times. At a wavelength of 274 nm, the ISP concentration in each sample was calculated using a UV–visible spectrophotometer. The cumulative percentage of ISP permeated and steady state flux was calculated three times and presented as mean ± SD.

### 2.7. In Vivo Evaluation of Optimized ILEP Formulation

#### 2.7.1. Animals

Twenty-four mature male albino rats (200–250 g body weight) were housed in an air-conditioned room with free access to food and water and a humidity range of 30–70%. After a week of eating an adapted diet, the rats were weighed and divided into five groups of six (n = 6). Before the study began, the mean body weight, blood glucose level, red blood cells (RBCs), white blood cells (WBCs), alanine aminotransferase (ALT), aspartate aminotransferase (AST), urea, and creatinine were all recorded for all groups. The in vivo experiment was approved by the ethics committee at Beni-Suef University in Egypt (BSU-IACUC: 022-413).

#### 2.7.2. Treatment Protocol

Twenty-four adult male albino rats were divided into two groups. A control negative group (n = 6) was given intraperitoneal injections of 0.9% saline solution and fed a normal diet. The remaining rats (n = 18) were fed a high-fat diet consisting of 6% cholesterol, 1% sodium cholate, 0.2% propyl thiouracil, and 5% refined sugar for 12 weeks to induce the f diabetes and atherosclerosis [32]. Blood glucose levels were monitored to ensure that diabetes was successfully induced [33]. Triglyceride and cholesterol levels were measured to verify that atherosclerosis had been successfully induced [34,35]. Rats were split into three groups of six at random after 12 weeks. The second group was given a 0.9% saline solution by gavage every day and acted as a positive control. The third group was given a free ISP (1 mg/kg) solution by gavage every day for 10 days [36]. The fourth group was given the optimized ILEP formulation (1 mg/kg) via the nasal route every day for 10 days. The rats’ weights and general behavior were recorded weekly during the experiment [35]. The second, third, and fourth groups received an intraperitoneal injection of a 0.1 mg/100 gm concentration of ketamine (90 mg/kg) and xylazine (5 mg/kg) to induce ischemic stroke. Blocking the primary blood supply to the brain was accomplished by occluding the common carotid and middle cerebral arteries with an intraluminal suture [37]. Sutures were removed after 2 h to allow reperfusion in the ischemic brain areas for 22 h; after this period, the rats were evaluated for neurobehavioral activity and slaughtered for histopathological examinations.

#### 2.7.3. Neurobehavioral Study

Each group’s rats were trained in the respective neurobehavioral task both before the start of the experiment and again after 22 h after reperfusion. We used a point system to quantify the severity of the behavioral issues [37,38]. 

By making use of the rat’s natural instinct to grasp a vertical metal grid while dangling by its tail, the grip strength test is used to quantify rat muscle force in vivo [33]. 

The flexion test is an evaluation used to detect neurological abnormalities in rats by watching their forelimb postures while suspended by their tails [37]. 

According to the method provided by Tabassum et al., spontaneous motor activity can be utilized to infer a rat’s neurological status [39]. 

The Morris water maze test, as reported by Ashafaq et al., is used to assess the cognitive–behavioral abilities of rats by watching how well they remember their location in the maze [37]. 

#### 2.7.4. Histopathology Study

At the end of the study, rats were euthanized by cervical dislocation after receiving an intraperitoneal injection of a 0.1 mg/100 gm concentration of ketamine (90 mg/kg) and xylazine (5 mg/kg). Blood was centrifuged to separate serum, and the levels of cholesterol, TG, and glucose were measured in accordance with the manufacturer [34,35].

Brain tissue samples (4–6 µm) were preserved by fixing them in 10% neutral buffer formalin, dehydrating them in ethyl alcohol, cleaning them in xylene, and finally embedding them in paraffin. These specimens were stained with hematoxylin and eosin for histological examination [34].

#### 2.7.5. Toxicity Studies

The rats’ weights were recorded from the beginning of the experiment to the day of the sacrifice [40]. Alterations in appearance and attitude caused by death and ageing were also seen [40]. Hemoglobin (Hb), RBC, mean corpuscular volume (MCV), mean corpuscular hemoglobin (MCH), mean corpuscular hemoglobin concentration (MCHC), WBCs, differential WBCs (neutrophil, lymphocyte, and monocyte), platelet count, creatinine, urea, AST, and ALT were all tested after the serum was separated [40]. 

### 2.8. Statistical Analysis

SPSS (version 22, Chicago, IL, USA) was used for statistical analysis, and the results were summarized as the mean ± SD (standard deviation), with significance determined by Tukey’s post hoc test at a *p*-value of 0.05.

## 3. Results

### 3.1. Experimental Design

This study used a Box–Behnken analysis design to successfully prepare and optimize ILEP formulations. Various PLEP formulations were prepared as blanks, and the findings indicated that the absorbance of all PLEP formulations was minimal and had an insignificant (*p*-value > 0.05) impact on the determination of the entrapment efficacy of ILEP formulations. Table 1 showed that the EE% and vesicle size were both fit to a quadratic model. The model was chosen because of its insignificant lack of fit, significant *p*-value, and high F-value. Furthermore, the model’s predicted R^2^ of 0.9981 and 0.9951 and adjusted R^2^ of 0.9986 and 0.9964 indicate that it may be used to interpolate EE% and vesicle size data, respectively, with high confidence. The model’s signal is strong enough for optimization, and it may be used to direct the design process with an adequate precision of 188.948 for EE% and 107.067 for vesicle size, respectively.

### 3.2. Characterization of ILEP Formulations

**Figure 1:**
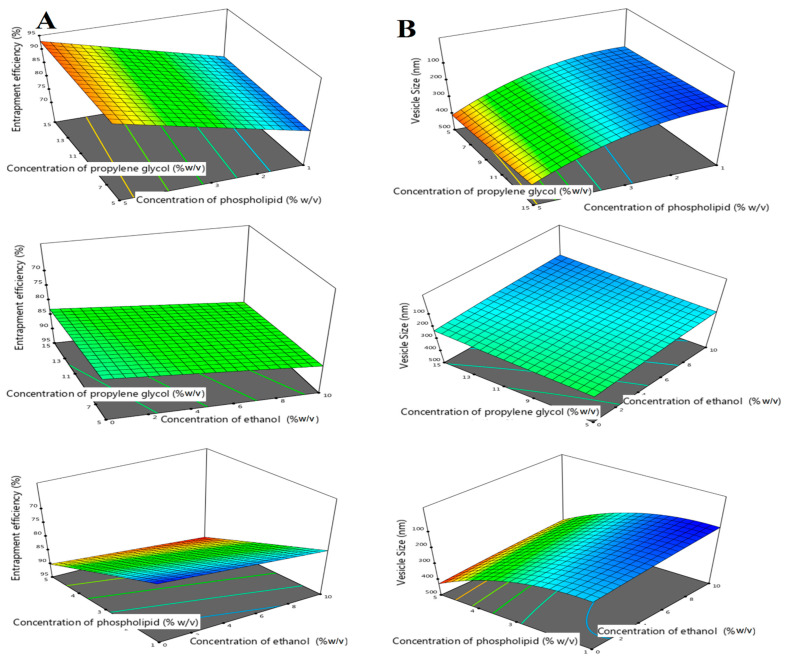
Three-dimensional response surface plot for the effect of concentration of propylene glycol (X1), concentration of phospholipid (X2), and concentration of ethanol (X3) on EE% (**A**) and vesicle size (**B**).

Table 2 shows that the ILEP formulations’ EE% ranged from 74.30 to 94.34% and particle size ranged from 141.77 to 424.57 nm. Equation (2) and Figure 1 show that propylene glycol, ethanol, and phospholipid have a positive (*p*-value < 0.0001) impact on EE%. Propylene glycol and ethanol have a negative (*p*-value < 0.0001) impact on particle size, while phospholipid has a positive (*p*-value < 0.0001) impact on particle size.

(2) $$\mathrm{Entrapment}\;\mathrm{Efficiency}\;=+84.30+0.8045\;X1+7.81\;X2+2.22\;X3+0.0425\;X1X2+\;0.0529\;X1X3\;-\;0.0509\;X2X3+0.0272\;{X1}^{2}+0.0606\;{X2}^{2}+0.0066\;{X3}^{2}$$

(3) $$\mathrm{Vesicle}\;\mathrm{size}\;=+215.44-15.60\;X1+112.89\;X2-28.39\;X3-1.37\;X1X2-0.45\;X1X3-0.283\;X2X3-\;2.31\;{X1}^{2}+64.22\;{X2}^{2}+3.22\;{X3}^{2}$$

### 3.3. Optimization of ILEP Formulation

After applying the criteria of smallest vesicle size and highest EE%, the Design Expert program suggested the optimized formulation. Because it has the highest desirability index (0.762), the formulation with 15% propylene glycol, 0.16% cholesterol, 10% ethanol, and 3.29% phospholipid was chosen as the optimum one. In vitro experiments of the optimized ILEP formulation showed that the predicted values of responses were accurate, with an average EE% of 88.56 ± 0.61% and a vesicle size of 189.62 ± 7.05 nm.

### 3.4. Characterization of Optimized ILEP Formulation

#### 3.4.1. DSC

**Figure 2:**
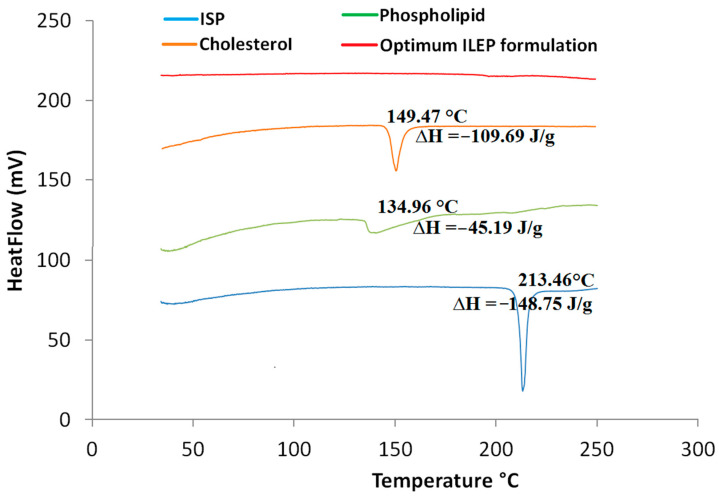
DSC thermograms of ISP, phospholipid, cholesterol, and optimized ILEP formulation; ∆H: enthalpy fusion.

Figure 2 shows that the endothermic peak of the ISP thermogram occurs at its melting point of 213.46 °C, the endothermic peak of the phospholipid thermogram occurs at its melting point of 134.96 °C, and the endothermic peak of the cholesterol thermogram occurs at its melting point of 149.47 °C. The thermogram of the optimized ILEP formulation showed that the peaks of ISP, phospholipid, and cholesterol disappeared.

#### 3.4.2. TEM

**Figure 3:**
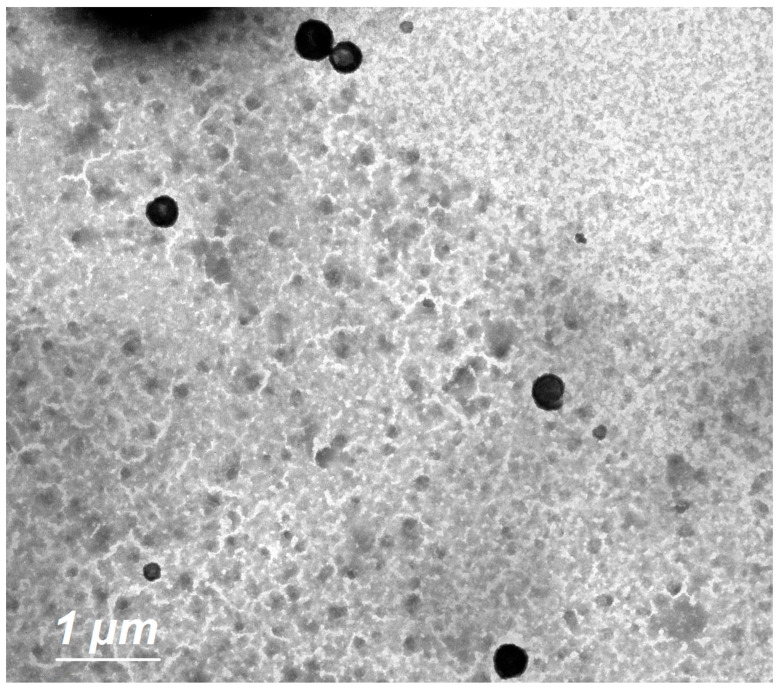
Surface morphology of optimized ILEP formulation by TEM at 4000× magnification; scale bar  =  1 µm.

The TEM image (Figure 3) revealed the formation of spherical vesicles appearing as black dots without aggregates.

#### 3.4.3. Zeta Potential and Size Distribution

With a PDI of 0.281, the optimized ILEP formulation showed that uniform vesicles were formed. With a zeta potential of −41.5 mV, the optimized ILEP formulation showed increased vesicle stability and decreased aggregation propensity of vesicles.

#### 3.4.4. In Vitro Release Study

**Figure 4:**
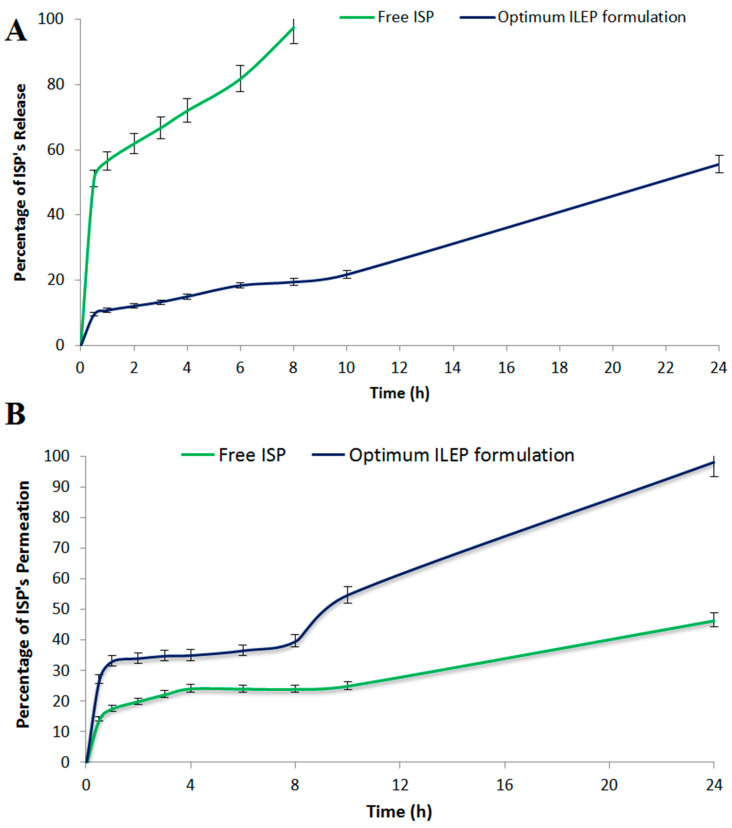
(**A**) In vitro release profile; (**B**) ex vivo drug permeation profile from the optimized ILEP formulation in comparison to free ISP.

The in vitro drug release profile from the optimized ILEP formulation compared to the free ISP is shown in Figure 4A. Using Student’s *t* test, the total amount of ISP released from the free ISP solution was significantly (*p*-value < 0.05) greater than the total amount of ISP released from the optimized ILEP formulation. A sustained release of 55.59% of ISP from the optimized ILEP formulation was observed at 24 h compared to that of free ISP with 97.42% at 8 h.

#### 3.4.5. Ex Vivo Skin Permeation Study

The ex vivo drug permeation profile from the optimized ILEP formulation compared to the free ISP is shown in Figure 4B. Using Student’s T test, the total amount of ISP permeated from the optimized ILEP formulation (1965.59 ± 83.24 µg/cm^2^) was significantly (*p*-value < 0.05) greater than the total amount of ISP permeated from the free ISP solution (1071 ± 70.63 µg/cm^2^). In addition, the flux (Jss) of the optimized ILEP formulation (58.47 ± 3.24 µg/cm^2^/h) was significantly (*p*-value < 0.05) higher than the flux of ISP from the free ISP solution (27.91 ± 2.47 µg/cm^2^/h) with an enhancement ratio of 2.094 after 24 h.

### 3.5. In Vivo Evaluation of Optimized ILEP Formulation

#### 3.5.1. Anti-Stroke Activity Measurement

**Figure 5:**
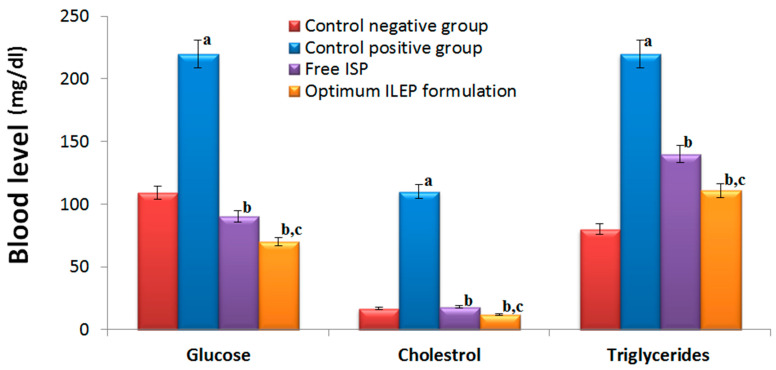
Serum glucose, cholesterol, and triglycerides of rats among different treatment groups. Significance: ^a^ *p*-value < 0.05 versus control negative group; ^b^ *p*-value < 0.05 versus control positive group; ^c^ *p*-value < 0.05 versus free ISP group.

Blood glucose, cholesterol, and triglyceride levels in the control positive group were significantly (*p*-value < 0.05) greater than those in the control negative group (Figure 5) by 2.02, 6.47, and 2.75 times, respectively, showing that the induction of diabetes and atherosclerosis in rats was successful. Upon treatment with free ISP and optimized ILEP formulation, blood glucose, cholesterol, and triglyceride levels were all found to be significantly (*p*-value < 0.05) lower than that of the control positive group, suggesting their antidiabetic and anti-atherosclerotic activities. Among treatment groups, the serum glucose, cholesterol, and TG levels of the optimized ILEP formulation group were the lowest with 68.18%, 89.09%, and 49.54%, respectively. Compared to the free ISP group, serum glucose, cholesterol, and triglyceride levels were shown to be significantly (*p*-value < 0.05) lower in the optimized ILEP formulation group by ratios of 1.15, 1.06, and 1.36, respectively.

**Figure 6:**
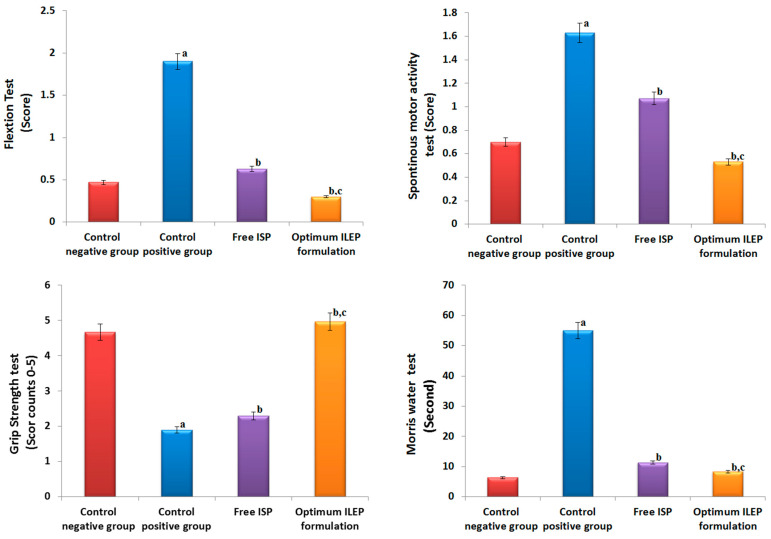
Effect on behavioral activities of rats among different treatment groups. Significance: a *p*-value < 0.05 versus control negative group; b *p*-value < 0.05 versus control positive group; c *p*-value < 0.05 versus free ISP group.

Ischemic stroke was successfully induced in rats, as shown in Figure 6. In the flexion and spontaneous motor activity tests, the control positive group had significantly (*p*-value < 0.05) higher activities compared to the control negative group by 4.04- and 2.33-fold, respectively. In the grip strength test, the control positive group had significantly (*p*-value < 0.05) lower activity compared to the control negative group by 2.45-fold. In the Morris water maze test, the control positive group took significantly (*p*-value < 0.05) longer time to locate the target quadrant compared to the control negative group by 8.69-fold. Upon treatment with free ISP and optimized ILEP formulation, flexion, spontaneous motor activities, grip strength, and time required to locate the target quadrant were all found to be significantly (*p*-value < 0.05) restored compared to that of the control positive group, suggesting their anti-ischemic stroke activity. Among treatment groups, the flexion, spontaneous motor activity, and time spent in the target quadrant of the optimized ILEP formulation group were the lowest with 84.21%, 67.48%, and 84.85%, respectively, and the grip strength of the optimized ILEP formulation was the highest by a ratio of 2.62%. Compared to the free ISP group, flexion, spontaneous motor activity, and time spent in the target quadrant were shown to be significantly (*p*-value < 0.05) lower in the optimized ILEP formulation group by a ratio of 26, 1.96, and 1.07, respectively, and the grip strength of the optimized ILEP formulation was shown to be significantly (*p*-value < 0.05) higher by a ratio of 2.16%.

**Figure 7:**
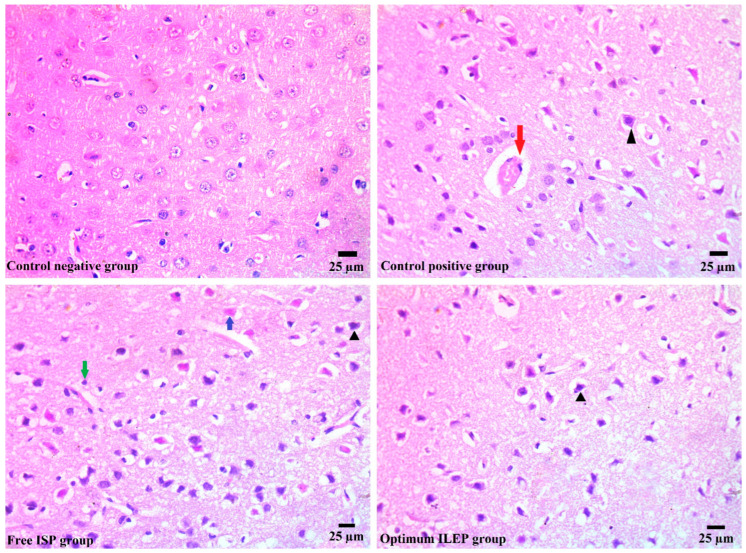
Histopathology images of brain tissue of all treated and untreated groups. Blue arrow: dead shrunken neurons, red arrow: edema, green arrow: glios, and arrowhead: pyknotic nuclei.

Ischemic strokes were successfully induced in rats, as shown in Figure 7. The photomicrographs from the control negative group showed a brain with a normal histological structure, but those from the control positive group showed congested blood capillaries swollen with edema (red arrow), along with dead shrunken neurons and pyknotic nuclei in some of them (arrowhead). Upon treatment with free ISP and optimized ILEP formulation, the congested blood capillaries, edema, dead shrunken neurons (blue arrow), and pyknotic nuclei were all found to be decreased compared to that of the control positive group, suggesting their anti-ischemic stroke activity. The optimized ILEP formulation group showed the highest activity compared to the free ISP group, which showed presence of gliosis (green arrow) and neuronal pyknosis (arrowhead).

#### 3.5.2. Toxicity Studies

Daily nasal treatment of an optimized ILEP formulation resulted in minimal toxicity or mortality in rats, and there were no behavioral changes compared to the control positive group. In the optimized ILEP formulation group, measurements showed a nonsignificant (*p* > 0.05) rise in body weight compared to the control negative group, indicating healthy growth. The hematological parameters such as Hb, RBC, MCV, MCH, MCHC, WBCs, neutrophil, lymphocyte, and monocyte and biochemical parameters such as creatinine, urea, AST, and ALT were not significantly different (*p* value > 0.05) between the optimized ILEP formulation group and the control negative group.

## 4. Discussion

With this study, the authors focused on developing an ILEP formulation for the treatment of ischemic stroke in diabetic patients. ILEP formulations are lipid vesicles composed of phospholipids, cholesterol, propylene glycol, and ethanol [19,21,41]. Phospholipids are vesicles’ fundamental components [20,21]. Cholesterol was included because it tightens up the spaces between the poorly packed lipid species in vesicular membranes, leading to better membrane integrity and drug entrapment [20,21]. Both propylene glycol and ethanol enhance the penetration of drugs through the nasal mucosa [21,22,41,42]. They reduce the rigidity of the lipid bilayer in the nasal barrier and boost its pliability, permeability, and fluidity. To boost vesicle concentration in the deep skin, propylene glycol, which is more viscous and hygroscopic than ethanol, may increase vesicle affinity to molecules in the water-based dermis layer [41]. As a result, our research incorporated the Box–Behnken design to investigate the effect of phospholipids, propylene glycol, and ethanol on EE% and vesicle size. Using an ANOVA test, *p*-value, lack of fit, F-value, predicted R^2^, adjusted R^2^, and adequate precision, Design Expert software determined which model gave the best fit [43]. The data analysis led to the selection of the quadratic model for EE% and vesicle size because it had a significant *p*-value, insignificant lack of fit, and highest values of F-value, predicted R^2^, and adjusted R^2^. Since the model’s appropriate precision is greater than 4, it is judged to be accurate enough for optimization. 

Analysis of the impact of each variable can be carried out with the help of the program’s coded polynomial equations and 3D surface graphs [31,44]. There was a statistically significant impact of propylene glycol, ethanol, and phospholipid on the EE%. Increased drug solubility from higher amounts of ethanol and propylene glycol causes more ISP to be entrapped and distributed uniformly throughout the vesicle. A higher quantity of phospholipid raises the total amount of lipid particles in each vesicle, making space for the ISP’s incorporation. Similar outcomes were reported by Akhtar et al., who enhanced the delivery of clotrimazole as an antifungal drug using ethosomes [45]. Phospholipid has a positive impact on particle size because higher concentrations of phospholipid led to the formation of thick, viscous bilayers that increased mass transfer resistances. Propylene glycol and ethanol have a negative impact on particle size because hydrocarbon chains of lipid vesicles can be penetrated by propylene glycol and ethanol, which may alter the net charge of the vesicles and lead to steric stabilization, hence decreasing their vesicle size. A previous study reported by Tawfik et al. agreed with these results [46]. Numerical optimization was used to estimate the optimal formulation’s composition following data analysis. Validity and applicability of the optimization design were confirmed by comparing the measured EE% and vesicle size of the optimized formulation to the predicted values supplied by the program.

Using DSC, we can measure the crystallinity of ISP and keep track of any interactions between ISP and the other ingredients in our optimized ILEP formulation [46]. The disappearance of the ISO peak in the optimized ILEP formulation suggested that the drug may have been molecularly dispersed or may have been present in an amorphous state and trapped in the lipid bilayer of the vesicles. The optimized ILEP formulation showed a low PDI and a homogenous vesicle distribution. The optimized formulation has a negative zeta potential value, which aids in vesicular stabilization. The electrostatic repulsion between vesicles is reduced by ethanol and phospholipid, making vesicles more stable when stored [23,47]. Propylene glycol, when coupled with ethanol, can improve vesicle stability by raising the formulation’s viscosity [41,42]. The TEM pictures verified that the vesicles were electrostatically stabilized and did not aggregate. An equilibrium solubility analysis was performed to determine the sink condition and the volume of dissolution media required for the release and permeation studies. We used 20 mL of phosphate-buffered (pH 6.8) solution because this volume is greater than the saturation solubility of ISP. Compared to free ISP, the release rate of the optimized ILEP formulation was much lower since phospholipid has a greater mass transfer resistance, and the ISP in the optimized ILEP formulation was deeply entrapped and distributed uniformly throughout the vesicle [23,46]. Ex vivo drug permeation studies have a strong correlation with the in vivo efficacy of nasal medication delivery systems [23,46]. Compared to free ISP, the flux of the optimized ILEP formulation was much higher because of the penetration efficiency of phospholipid, ethanol, and propylene glycol, which make the skin’s lipid bilayer more flexible, diffusive, and fluid [21,41,42,45]. 

A stroke occurs when there is a blockage in the blood supply to the brain, usually caused by thrombosis or embolism [1,3]. Hyperglycemia, which is characteristic of diabetes mellitus, contributes to the development of endothelial dysfunction, dyslipidemia, and insulin resistance, all of which increase the risk of stroke [1,2,3]. Due to its ability to stimulate inflammatory cell infiltration and phagocytosis, as well as plaque calcification after endothelial injury, coexisting atherosclerosis has been shown to have superior predictive value for stroke [48,49,50,51,52]. Therefore, we accelerated an experimental model of ischemic stroke in rats by causing diabetes and atherosclerosis in them with a high-fat and sugar-rich diet over the course of 12 weeks [33,50]. Serum glucose, cholesterol, and triglyceride levels were all higher in the control positive rats compared to the control negative rats, demonstrating that we had successfully induced diabetes and atherosclerosis in rats. Observing severe behavioral abnormalities such as increased flexion, spontaneous motor activity, and memory deficits in the control positive rats compared to the control negative rats demonstrated that we had successfully induced strokes in the rats. Histopathology verified the successful induction of strokes in the rats by demonstrating the presence of congestive blood vessels, edema, and dead, shrunken neurons in the control positive rats. The results of improved neurological behavior and decreased edema, dead shrunken neurons, and vascular congestion reflected the anti-stroke activities of both free ISP and the optimized ILEP formulation because ISP is an effective beta-adrenergic agonist that improves circulation to the vulnerable penumbral region after ischemia and prevents the evolution of the infarct core [7,8,9]. Furthermore, ISP has strong α1-adrenoreceptor antagonist action, which may increase neuroprotection by increasing cerebral vasodilation and decreasing vasoconstriction in ischemic damage [5,6]. The optimized ILEP formulation group showed the highest activity compared to the free ISP group because of the penetration efficiency of phospholipid, ethanol, and propylene glycol, which make the skin’s lipid bilayer more flexible, diffusive, and fluid [21,41,42,45]. Furthermore, optimized ILEP improved the sustainability and targeting of ISP by functioning as a reservoir at the site of application and releasing the drug slowly over time [23,46,53]. 

Toxicology studies on animals were conducted to better understand the potential hazards of nasal administration of the optimized ILEP formulation and to identify any noticeable adverse effects. Compared to the control negative group, all rats administered the optimized ILEP formulation showed no signs of illness or death and no significant differences in body weight, demonstrating its normal growth. Due to the lack of significant fluctuation in RBC and WBC, it was determined that the nasal delivery of the optimized ILEP formulation had no effect on the immune system, erythropoiesis, or the morphology or osmotic fragility of red blood cells. Liver and kidney functions were unaffected by nasal delivery of the optimized ILEP formulation since there was no significant difference in the levels of serum ALT, AST, creatinine, and urea. These results indicated that the optimized ILEP formulation had no detectable influence on normal growth, indicated no organs were negatively affected, and showed no evidence of toxicity when administered via the nasal route.

## 5. Conclusions

With this study, the authors focused on developing an ILEP formulation for the treatment of ischemic stroke in diabetic patients. The release studies verified the sustained release potential of the optimized ILEP formulation, demonstrating its ability to increase the biological half-life and slow the elimination of ISP. Furthermore, the in vivo studies verified the improved neurological behavior and decreased edema, dead shrunken neurons, and vascular congestion of the rats treated with the optimized ILEP formulation, demonstrating its anti-stroke activity. In conclusion, our study found that treatment with an optimized ILEP formulation prevented the initiation and severity of stroke, especially in diabetic patients.

## Figures and Tables

**Table 1:** The independent and dependent variables of ILEP formulations.

Independent Variables	Coded Values							
	−1		0			+1		
X_1_: concentration of propylene glycol (%)	5		10			15		
X_2_: concentration of phospholipid (%)	1		3			5		
X_3_: concentration of ethanol (%)	0		5			10		
Dependent Variables	Model	Lack of Fit	F-value		*p*-value		Constraints	
Y_1_: Entrapment efficiency (%)	Quadratic	0.8584	3499.20		<0.0001		Maximize	
Y_2_: Vesicle size (nm)	Quadratic	0.4792	1337.81		<0.0001		Minimize	

**Table 2:** Compositions and responses of ILEP formulations.

Run	Factors Levels in Actual Values			Responses (n = 3)	
	X_1_: Concentration of Propylene Glycol (%)	X_2_: Concentration of Phospholipid (%)	X_3_: Concentration of Ethanol (%)	Entrapment Efficiency (% ± SD)	Vesicle Size (nm ± SD)
F1	10	1	10	78.84 ± 0.22	141.77 ± 4.90
F2	10	1	0	74.30 ± 0.30	195.53 ± 4.80
F3	10	3	5	84.27 ± 0.25	217.33 ± 3.79
F4	5	3	10	85.66 ± 0.24	203.00 ± 3.61
F5	5	3	0	81.32 ± 0.25	261.33 ± 5.51
F6	15	3	0	82.90 ± 0.19	230.60 ± 3.67
F7	5	1	5	75.85 ± 0.26	179.83 ± 7.15
F8	10	3	5	84.33 ± 0.22	215.67 ± 7.02
F9	10	5	10	94.34 ± 0.24	369.67 ± 4.51
F10	15	5	5	93.01 ± 0.21	372.13 ± 6.74
F11	15	1	5	77.30 ± 0.22	151.80 ± 9.82
F12	15	3	10	87.46 ± 0.27	170.47 ± 3.88
F13	10	5	0	90.00 ± 0.19	424.57 ± 5.52
F14	5	5	5	91.40 ± 0.27	405.67 ± 7.02
F15	10	3	5	84.31 ± 0.24	213.33 ± 4.51

SD, standard deviation.

## Data Availability

The corresponding author will make the datasets used in this study available upon reasonable request.
